# Ultrasound-Guided Pharmacopuncture Targeting the Myotendinous Junction for Refractory Common Extensor Tendinopathy: A Case Series

**DOI:** 10.3390/diagnostics16172755

**Published:** 2026-08-28

**Authors:** Taeseok Ahn, Jihyun Moon, Myungjin Oh, Junyoung Oh, Jung-Yun Ahn, Daeook Lee, Jongha Lee, Changyoung Park, Dong-Woo Lim

**Affiliations:** 1 VARO Korean Medicine Clinic, Seoul 07714, Republic of Korea; truenote87@gmail.com (T.A.); lunarjihyun@gmail.com (J.M.); 2 Department of Acupuncture and Moxibustion Medicine, School of Korean Medicine, Pusan National University, Yangsan 50612, Republic of Korea; kkomc@hanmail.net (M.O.); ojy6362@naver.com (J.O.); 3 Dasram Cancer Care Hospital, Goyang 10250, Republic of Korea; rurujy77@naver.com; 4 SAMSUNG Korean Medicine Clinic, Pohang 37788, Republic of Korea; woogi0418@gmail.com; 5 BAREUNBUBU Korean Medicine Clinic, Naju 58325, Republic of Korea; thebell8888@gmail.com; 6 Samsong Myeongmun Korean Medicine Clinic, Goyang 10571, Republic of Korea; taekyo1202@gmail.com; 7 Department of Diagnostics, College of Korean Medicine, Dongguk University, Goyang 10326, Republic of Korea

**Keywords:** lateral elbow tendinopathy, acupuncture injection therapy, myotendinous junction intervention, tissue hydrodissection, musculoskeletal ultrasonography, retrospective case series

## Abstract

**Background**: Lateral epicondylalgia (LE) is a prevalent musculoskeletal condition, and a substantial proportion of patients fail to respond to conservative treatment. The myotendinous junction (MTJ) of the common extensor tendon (CET) harbors dense nociceptive nerve endings yet remains an underexplored target in pharmacopuncture practice. **Case Presentation**: In this retrospective case series, three patients with refractory LE (duration 1.5–3 years, with prior treatments including corticosteroid injection, NSAIDs, physical therapy, and prolotherapy) received a structured multicomponent ultrasound-guided pharmacopuncture protocol, of which MTJ-targeted hydrodissection with placenta pharmacopuncture constituted the primary component, administered over ten sessions. A structured three-stage protocol was applied, incorporating placenta pharmacopuncture at the MTJ, Eohyeol pharmacopuncture at LI11, and dry needling at the CET enthesis. **Results**: All three patients achieved clinically meaningful improvements in PRTEE scores (Case 1: 64→13, −79.7%; Case 2: 73→38, −47.9%; Case 3: 55→11.5, −79.1%), exceeding the MCID threshold of 37% reduction. Qualitative sonographic changes were observed at follow-up assessments conducted 103–270 days after treatment completion. No adverse events were observed. **Conclusions**: A structured multicomponent ultrasound-guided pharmacopuncture protocol may represent a feasible non-surgical approach for patients with refractory LE. Controlled prospective studies are warranted.

## 1. Introduction

Lateral epicondylalgia (LE), commonly referred to as tennis elbow, is a degenerative tendinopathy of the common extensor tendon (CET) at the lateral epicondyle of the humerus, with a prevalence of approximately 1–3% in the general population [1]. It predominantly affects individuals in their fourth to fifth decades of life and is strongly associated with repetitive upper limb use in occupational settings [2,3]. Although conservative treatments including corticosteroid injections, nonsteroidal anti-inflammatory drugs (NSAIDs), and physical therapy are the mainstay of initial management, a significant proportion of patients experience persistent symptoms or recurrence, and some are ultimately referred for surgical intervention [4,5].

Identifying the optimal injection site is of crucial importance given the multifactorial pathophysiology and chronic progressive nature of LE. From a pathophysiological perspective, the CET enthesis has traditionally been regarded as the primary site of tendinopathic change [6]. A case–control study of extensor carpi radialis brevis (ECRB) tendon samples demonstrated increased inflammatory cell infiltration and inflammatory marker expression in patients with chronic lateral epicondylitis compared with controls, suggesting that inflammatory mechanisms may coexist with degenerative changes in this condition [2]. Meanwhile, a cadaveric study by Evans et al. demonstrated that injection at the CET enthesis successfully spread injectate through 97% of the cross-sectional area, supporting the CET as a classical infiltration site for ultrasound-guided interventions in the management of LE [7]. Clinical trials report a heterogeneous selection of injectate volumes and delivery techniques, with systematic reviews finding no clear consensus.

However, recent evidence highlights the potential importance of the myotendinous junction (MTJ) as an additional pain source, given the dense distribution of nociceptive nerve endings within this region [8]. The MTJ harbors four types of mechanoreceptive nerve endings—Ruffini corpuscles, Pacini corpuscles, Golgi tendon organs, and free nerve endings—of which the latter function as nociceptors [9]. Furthermore, a preliminary clinical report by Rosales et al. suggested that MTJ-targeted injection may offer advantages over enthesis-targeted injection in the management of LE, although this observation requires validation in controlled studies [8]. However, existing local injection therapy studies for LE have predominantly targeted the CET enthesis or peritendinous space [10], and reports of MTJ-targeted pharmacopuncture are exceedingly rare in the literature.

Ultrasound (US) scanning enables precise, real-time visualization of the musculoskeletal and surrounding neurovascular structures [11]. US has become widely adopted in both the diagnosis and image-guided intervention of musculoskeletal disorders. A representative application of US in a clinical setting is US-guided needling therapy (acupuncture) or pharmacopuncture, facilitating accurate needle placement and reducing procedural risk [12]. With increasing accessibility of ultrasound equipment and growing recognition of its clinical utility, reports of US-guided pharmacopuncture in musculoskeletal conditions have been accumulating.

The use of hydrodissection—a technique in which the injectate is used to mechanically dissect tissue planes—has been proposed as a means of improving the perineural and peritendinous microenvironment, in addition to delivering pharmacological agents [13]. Combined with US-guidance techniques, hydrodissection provides a precise and efficient strategy for addressing tissue pathology and alleviating local pain in LE [14]. However, detailed intervention protocols and high-quality clinical evidence for US-guided pharmacopuncture targeting specific musculoskeletal conditions remain scarce.

In this context, we report three cases of refractory LE in which ultrasound-guided pharmacopuncture with sequential hydrodissection targeting the MTJ of the CET was performed, resulting in clinically meaningful improvements in pain and function.

## 2. Case Presentation

### 2.1. Patient Characteristics and Medical History

Refractory LE was operationally defined as persistent lateral elbow pain of at least 12 months’ duration that had not responded to a minimum of one prior evidence-based conservative treatment, including but not limited to corticosteroid injection, NSAIDs, physical therapy, or prolotherapy.

Case 1 was a 62-year-old woman who had experienced lateral elbow pain for approximately 1.5 years. She worked as a butcher, regularly handling heavy cuts of meat with repetitive cutting motions, and reported exacerbation of symptoms during a recent period of increased workload. She had been diagnosed with a partial tear of the CET at an orthopedic clinic and had undergone corticosteroid (triamcinolone) injection, which provided no lasting relief. She was taking no medications at the time of the initial visit.

Case 2 was a 54-year-old man who had experienced lateral elbow pain for approximately 2 years. He worked as a plumber, regularly gripping and operating heavy equipment. He had been diagnosed with a partial tear of the CET and had received corticosteroid injection, NSAIDs (naproxen 250 mg), and physical therapy at an orthopedic clinic without sustained improvement. Arthroscopic debridement had been recommended but declined by the patient. He had discontinued NSAIDs one month prior to the initial visit due to gastrointestinal discomfort and was taking no medications at presentation.

Case 3 was a 48-year-old man who had experienced lateral elbow pain for approximately 3 years. He worked as a chef, regularly performing repetitive wrist extension and forearm rotation. He had received prolotherapy injections at a secondary-care hospital without meaningful improvement and had taken medical leave from work. He had a history of hypertension and was taking an antihypertensive agent.

The three cases were identified through retrospective review of medical records from the participating clinics (June 2021–August 2025) and represent all consecutive patients who met the predefined inclusion criteria during this period. No patients who completed the full treatment protocol were excluded.

This case series adheres to the CARE guidelines (see Supplementary Material File S1 for the completed CARE checklist). The study was approved by the Institutional Review Board of Dongguk University (DGU IRB 20260070, date of approval: 23 July 2026). The clinical treatments were performed as routine clinical care prior to the initiation of this retrospective study. IRB approval was obtained retrospectively for the purpose of medical record review and case report publication. The IRB waived the requirement for prospective research participation consent, as the study involved retrospective review of de-identified records. Written informed consent for publication of clinical details and images was obtained from all patients.

### 2.2. Clinical Findings

In all three cases, physical examination revealed reproduction of lateral elbow pain on Cozen’s test (resisted wrist extension). Focal tenderness was elicited not only at the CET enthesis but also on direct compression of the MTJ. All patients demonstrated negative findings on the finger extension test, resisted forearm supination test, and passive forearm pronation test, thereby reducing clinical suspicion for radial tunnel syndrome and posterior interosseous nerve entrapment. Cervical Spurling’s test was negative in all cases, making cervical radiculopathy an unlikely contributing factor in the clinical context of each case.

### 2.3. Diagnostic Assessment

Diagnosis was established through a combination of clinical history, physical examination, and ultrasound imaging. All examinations and procedures were performed by a single Korean medicine practitioner with over 13 years of clinical experience, holding the Registered in Musculoskeletal Sonography (RMSK) credential administered by the Alliance for Physician Certification & Advancement (APCA).

Ultrasound imaging was performed using a GE LOGIQ system. Case 1 was examined using a GE LOGIQ Fortis with an L6-24 linear transducer (GE HealthCare, Seongnam, Republic of Korea); Cases 2 and 3 were examined using a GE LOGIQ S8 with an ML 6-15 linear transducer (GE HealthCare, Seongnam, Republic of Korea). The same system and transducer were used for each individual patient at all time points, including baseline and follow-up. For diagnostic scanning, the patient’s elbow was flexed to 90° and the transducer was initially positioned longitudinally (long-axis view), centered over the LI11 acupoint corresponding to the extensor carpi radialis longus, and then swept across the full extent of the CET enthesis. Doppler scanning was performed using standardized settings before each treatment session to identify the radial nerve branches and the recurrent radial artery and to minimize the risk of neurovascular injury during needle insertion. For the intervention, the transducer was rotated to a transverse (short-axis) orientation at the level of the MTJ to enable real-time visualization of the target structures, including the extensor digitorum communis (EDC), extensor carpi radialis brevis (ECRB), supinator (SUP), and radius (R).

Sonographic findings in all three cases included hypoechoic changes at the CET, consistent with tendinopathy or partial tear. Prior to each procedure, the branches of the radial nerve were identified using B-mode ultrasonography, and Doppler scanning was performed to locate the recurrent radial artery to minimize the risk of neurovascular injury during needle insertion. In all three cases, sonographic assessment of the radiohumeral joint was performed during the diagnostic examination. No evidence of joint effusion, synovial thickening, intra-articular loose bodies, or cartilage irregularity was identified. Therefore, articular pathology of the lateral compartment was considered unlikely as a primary pain generator in all cases. The diagnostic ultrasound examination was performed in accordance with the ESSR Musculoskeletal Ultrasound Technical Guidelines for the elbow, including longitudinal and transverse assessment of the common extensor tendon, lateral collateral ligament complex, radiohumeral joint, and annular ligament. All sonographic findings reported in this series represent qualitative descriptive observations made by the treating practitioner in the course of routine clinical care and should be interpreted within this context.

### 2.4. Therapeutic Intervention

Patients were positioned supine with the elbow flexed to 90° and the forearm resting on the chest. The ultrasound transducer was wrapped with a sterile Tegaderm Film (3M, St. Paul, MN, USA), and the injection site was disinfected with a 10% povidone–iodine swab stick (Green Pharmaceutical, Seoul, Republic of Korea).

The treatment protocol consisted of three components targeting distinct pathological features of lateral epicondylalgia—myofascial dysfunction at the MTJ, regional pain at LI11, and CET enthesis pathology—administered according to a fixed schedule across a treatment course of 10 sessions: Stage 1 (MTJ hydrodissection), performed at every visit (10 sessions in total); Stage 2 (LI11 pharmacopuncture), administered only at the first and second visits (2 sessions in total); and Stage 3 (dry needling at the CET enthesis), administered only at the ninth and tenth visits (2 sessions in total).

The three-stage protocol described herein was developed and standardized by the primary treating practitioner (T.A.) prior to its application in Case 1 (2021) and was applied without modification across all three cases. All treatment procedures in all three cases were performed by the same practitioner (T.A.). Treatment was administered three sessions per week over approximately three to four weeks (total: 10 sessions), with each session lasting approximately 30 min.

**Figure 1:**
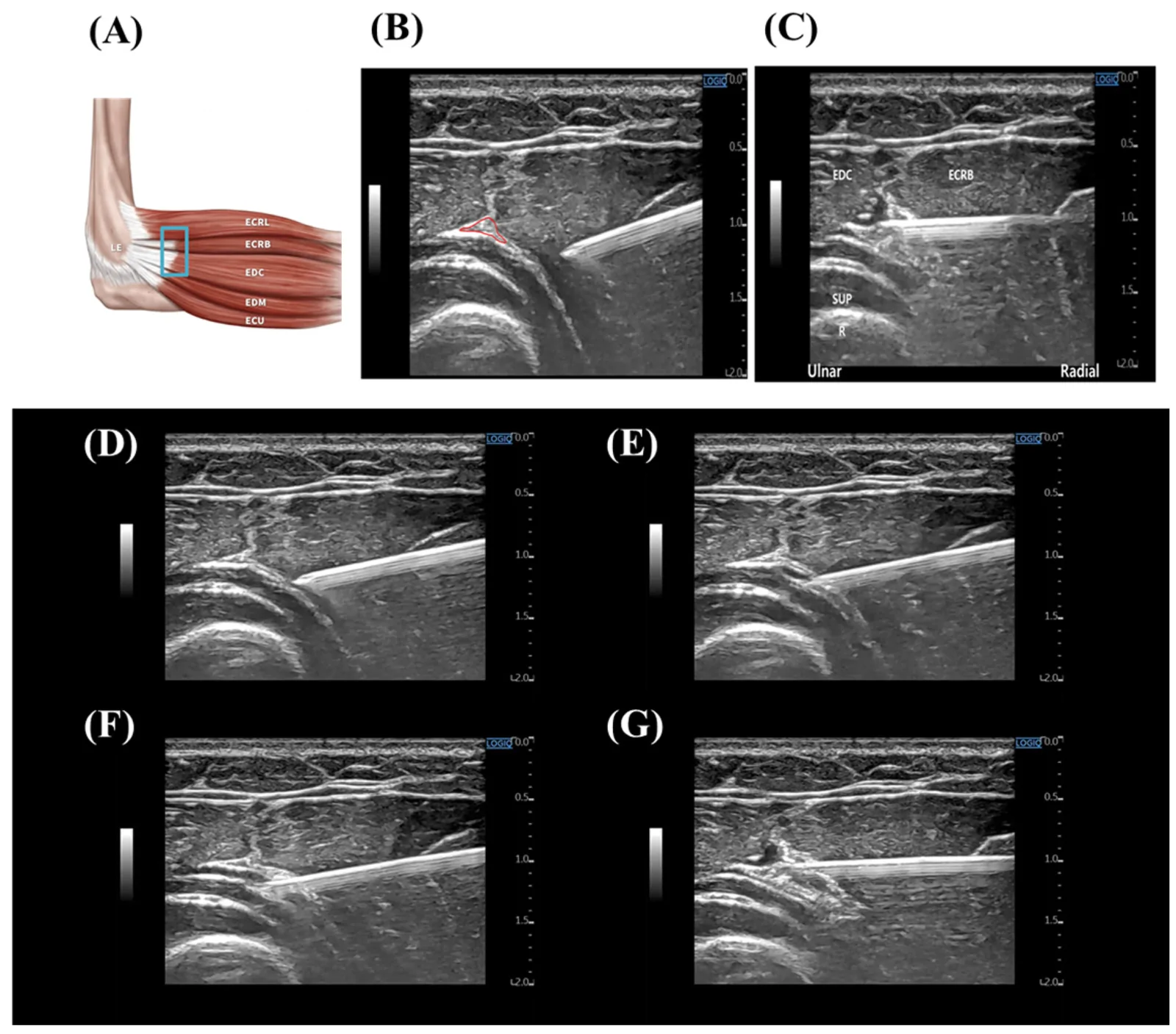
(**A**) Anatomical illustration of the right lateral elbow in 90° flexion, showing the extensor musculature and the transducer placement site (cyan box) at the level of the common extensor tendon myotendinous junction. (**B**) Pre-procedural transverse (short-axis) ultrasound image at the level of the myotendinous junction of the common extensor tendon, demonstrating the target anatomy prior to needle insertion. A hypoechoic lesion at the myotendinous junction is delineated (red triangle). The anatomical illustration indicates the probe placement site (cyan box). (**C**) Intra-procedural transverse (short-axis) ultrasound image obtained during pharmacopuncture delivery, showing the needle advanced to the myotendinous junction via a radial in-plane approach. Key anatomical landmarks are labeled: extensor digitorum communis (EDC), extensor carpi radialis brevis (ECRB), supinator (SUP), and radius (R). (**D**) Pre-injection image confirming needle positioning. (**E**) Intra-procedural image showing the needle advanced to the inferior aspect of the myotendinous junction with the bevel oriented upward (bevel-up), enabling hydrodissection of the ECRB aponeurosis. (**F**) Intra-procedural image demonstrating further hydrodissection performed within the target plane. (**G**) Intra-procedural image demonstrating needle repositioning (bevel-down) for hydrodissection of the superior myotendinous junction (see Supplementary Material File S2). CET, common extensor tendon; ECRL, extensor carpi radialis longus; ECU, extensor carpi ulnaris; EDC, extensor digitorum communis; ECRB, extensor carpi radialis brevis; EDM, extensor digiti minimi; LE, lateral epicondyle; SUP, supinator; R, radius.

Stage 1: MTJ hydrodissection with placenta pharmacopuncture (every visit; ten sessions in total). The MTJ was identified on transverse ultrasound imaging as the zone of transition between the hypoechoic muscle belly and the echogenic tendinous component of the EDC and ECRB (Figure 1A–C). A point of maximal tenderness on probe compression, corresponding to an Ashi point within the Large Intestine meridian muscle region, was marked with a surgical marking pen. Using an L6-24 linear transducer and a 27-gauge, 38 mm needle, 2 mL of placenta pharmacopuncture solution (prepared by the Seongnam external herbal dispensary of Jaseng Hospital of Korean Medicine) was administered to the MTJ region via a radial side in-plane approach under real-time ultrasound guidance, delivered in two sequential steps (Figure 1D–G):

Step 1 (inferior hydrodissection): With the needle bevel oriented upward, the needle tip was advanced to the inferior aspect of the MTJ. The injectate was deposited along the subtendinous plane to achieve hydrodissection between the tendinous components and the surrounding fascial tissue.

Step 2 (superior hydrodissection): The needle was repositioned with the bevel oriented downward to target the superior aspect of the MTJ, and the remaining injectate was deposited to achieve hydrodissection of the supratendinous plane.

Stage 2: Jungsong-eohyeol pharmacopuncture at LI11 (first and second visits only; two sessions in total). This intervention targeted Quchi (LI11), a commonly used acupoint located near the lateral elbow. Ultrasound guidance was performed using an ML6-15 linear transducer, and a 27-gauge, 38 mm needle was used. A low-concentration Eohyeol pharmacopuncture solution was prepared by diluting 2 mL of Eohyeol pharmacopuncture solution (AJ Korean Medicine Pharmacy, Seoul, Republic of Korea) in 50 mL of normal saline. A total of 3 mL of the diluted solution was injected into the deep fascial plane on the superficial surface of the CET enthesis under ultrasound guidance.

Stage 3: Dry needling at the CET enthesis (ninth and tenth visits only; two sessions in total). This intervention targeted the CET enthesis at the lateral epicondyle. Using an L6-24 linear transducer and a 27-gauge, 25 mm needle, ultrasound-guided dry needling was performed at the enthesis. A periosteal pecking technique was applied to mechanically stimulate the enthesis and adjacent periosteal tissue.

Standard aseptic technique was maintained throughout all procedures. The operator wore sterile gloves, and the injection site was disinfected with 10% povidone-iodine solution. Rather than applying ultrasound gel, the 10% povidone–iodine solution itself was used as the acoustic coupling agent on the probe footprint within the disinfected field to maintain sterility and optimize acoustic contact. All needles, syringes, and injectate vials were single-use. After the procedure, patients were monitored for immediate adverse reactions.

#### Rehabilitation Exercise

**Figure 2:**
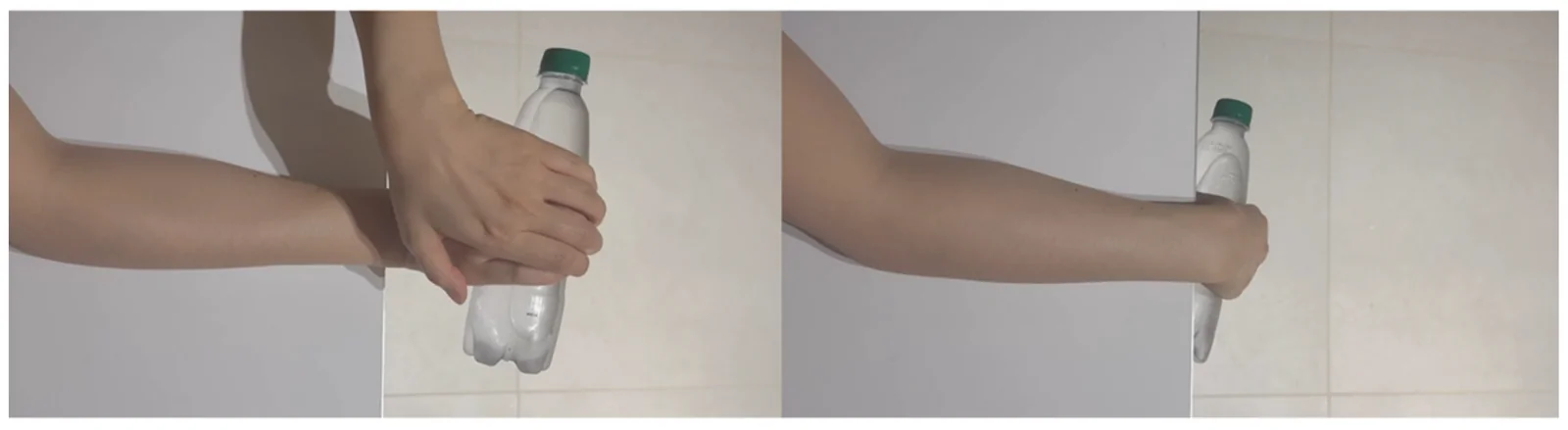
Home-based eccentric exercise protocol for the wrist extensor musculature. (**Left**) Starting position: the wrist is passively lifted into extension using the contralateral hand while gripping a water bottle (approximately 350 g). (**Right**) Eccentric phase: the wrist is lowered slowly over 3 s under controlled eccentric contraction of the wrist extensor musculature, without bouncing at the end range. The exercise was performed in three sets of 15 repetitions, twice daily. If pain exceeded 3/10 on a numeric rating scale during loading, isometric wrist extension in the neutral position was substituted.

Beginning at the ninth treatment session, all three patients were instructed in a home-based eccentric exercise program targeting the common extensor musculature, which was continued independently beyond the in-clinic treatment period and maintained through to the date of follow-up sonographic assessment (Figure 2). The exercise was performed using a handheld water bottle as a resistance implement.

The eccentric exercise was performed with the forearm resting on a table edge, where the wrist was lifted into extension using the contralateral hand and lowered slowly over 3 s without bouncing. It was performed in three sets of 15 repetitions, twice daily, using a water bottle as resistance (initial weight approximately 350 g, progressed incrementally as tolerated). A pain-monitoring rule was applied: exercise was continued if pain remained at or below 3/10 (tolerable pain) on a numeric rating scale and substituted with isometric wrist extension in neutral if pain exceeded this threshold. Adherence was assessed verbally at each clinic visit; all three patients reported consistent daily exercise performance throughout the treatment and follow-up.

### 2.5. Outcome Measures

Clinical outcomes were assessed using the Patient-Rated Tennis Elbow Evaluation (PRTEE), a validated 15-item patient-reported outcome measure that quantifies pain and functional disability associated with lateral elbow tendinopathy [15]. In the present series, the validated Korean-language version of the PRTEE was administered, the reliability and validity of which have been established by a previous report [15]. Clinically meaningful improvement was defined as a 37% or greater reduction from baseline, per the MCID established by Poltawski et al. [16].

The PRTEE was self-completed by each patient in paper form and collected by the treating practitioner at baseline (prior to the first session) and upon completion of the tenth session. As the treating practitioner also collected the outcome measure, assessor blinding was not performed, which represents a limitation of this retrospective series.

Adverse events were assessed at each treatment session through standardized verbal inquiry by the treating practitioner, covering local reactions (pain, bruising, swelling, infection), systemic reactions (dizziness, nausea, anaphylaxis), and delayed events reported since the prior session.

### 2.6. Case 1—Ultrasound Findings and Clinical Outcomes

**Figure 3:**
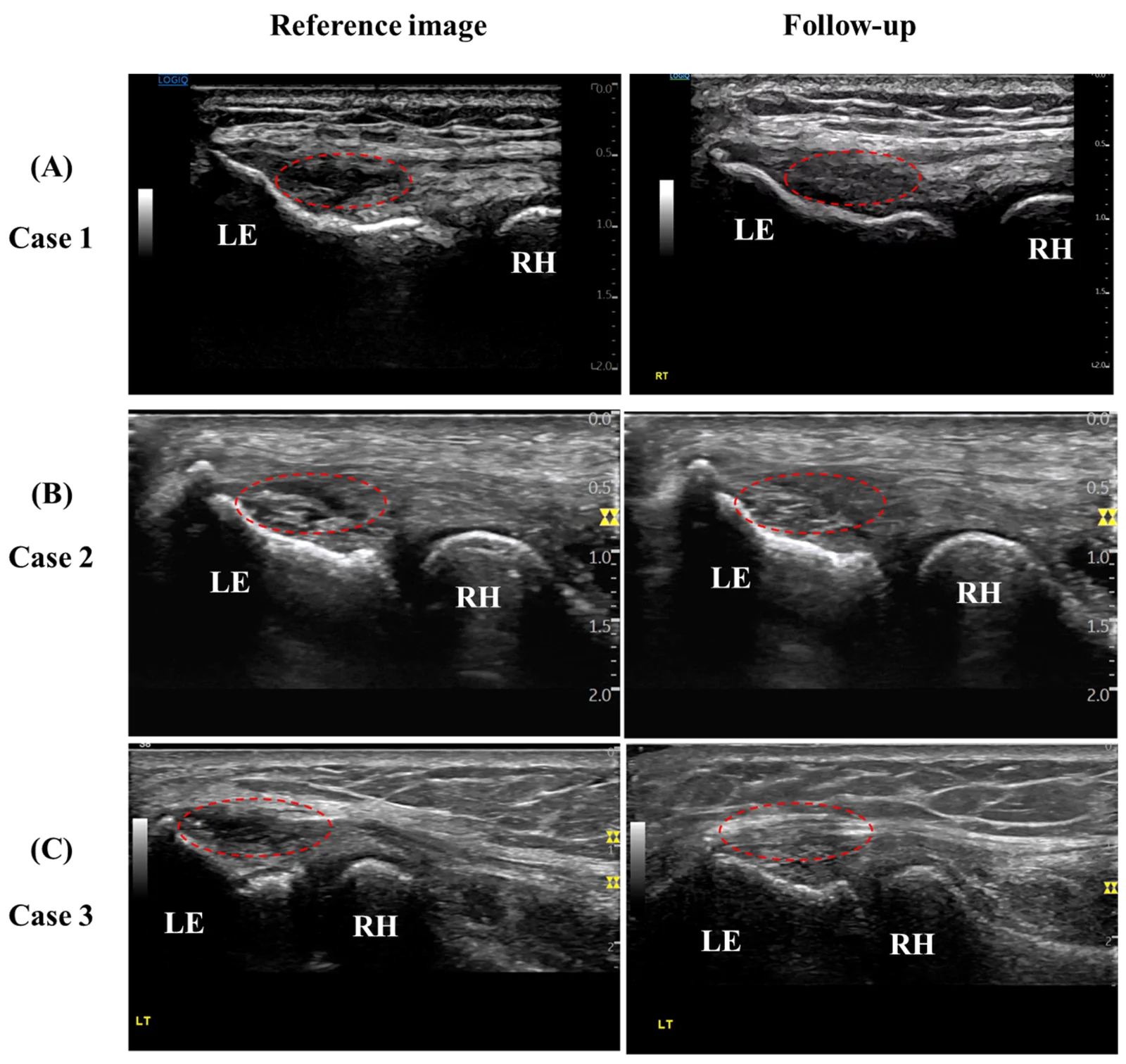
Longitudinal ultrasound images of the common extensor tendon at the lateral elbow: pre-treatment (Cases 1 and 2) or at treatment completion (Case 3)/(**left**) and follow-up (**right**). (**A**) Case 1. (**Left**): Hypoechoic partial tear at the CET enthesis with disrupted fibrillar echotexture. (**Right**): Follow-up at 114 days after treatment completion, showing increased echogenicity. (**B**) Case 2. (**Left**): Hypoechoic partial tear at the CET enthesis with bony irregularity at the lateral epicondyle. (**Right**): Follow-up at 103 days after treatment completion, showing partial resolution of the hypoechoic defect. (**C**) Case 3. (**Left**): Residual hypoechoic changes at the CET at treatment completion. (**Right**): Follow-up at 270 days after treatment completion, showing marked reduction in hypoechoic area with near-complete restoration of fibrillar echotexture. Image interpretation was performed by the treating practitioner, who was not blinded to treatment stage or clinical outcome. CET, common extensor tendon. LE, lateral epicondyle; RH, radial head.

On pre-treatment longitudinal ultrasound, a hypoechoic partial tear was identified at the CET enthesis with disruption of the normal fibrillar echotexture. On the PRTEE administered at baseline (14 April 2025), the patient scored 64 points. Following ten sessions of ultrasound-guided MTJ pharmacopuncture, the PRTEE score decreased to 13 points (7 May 2025), representing a reduction of 51 points (−79.7%). Follow-up ultrasound conducted 114 days after treatment completion (6 August 2025) demonstrated reduction in the hypoechoic area with partial restoration of fibrillar echotexture at the previously affected site. No recurrence of symptoms was reported at follow-up (Figure 3A, Table 1, Table 2 and Table 3).

### 2.7. Case 2—Ultrasound Findings and Clinical Outcomes

Pre-treatment longitudinal ultrasound demonstrated a hypoechoic partial tear at the CET enthesis with associated bony irregularity at the lateral epicondyle. The baseline PRTEE score was 73 points (18 July 2022). Following completion of ten treatment sessions, the PRTEE score decreased to 38 points (10 August 2022), a reduction of 35 points (−47.9%). Although the degree of improvement was comparatively modest relative to the other cases, this patient presented with the most severe baseline findings, having been referred for arthroscopic surgery. Follow-up ultrasound at 103 days post-completion (21 November 2022) demonstrated partial reduction in hypoechoic signal with modest improvement in fibrillar definition. The patient did not proceed with the previously recommended surgery (Figure 3B, Table 1, Table 2 and Table 3).

### 2.8. Case 3—Ultrasound Findings and Clinical Outcomes

Longitudinal ultrasound obtained at treatment completion demonstrated residual hypoechoic changes in the CET consistent with tendinopathy, as a pre-treatment baseline image was not available for retrospective retrieval. The baseline PRTEE score was 55 points (11 June 2021). Following ten treatment sessions, the PRTEE score decreased to 11.5 points (30 June 2021), a reduction of 43.5 points (−79.1%). The patient returned to work during the treatment period. Longitudinal ultrasound obtained at 270-day follow-up (8 March 2022) demonstrated marked reduction in hypoechoic area with near-complete restoration of fibrillar echotexture. No recurrence was reported at the time of follow-up (Figure 3C, Table 1, Table 2 and Table 3).

### 2.9. Patient Perspective

The following accounts were compiled retrospectively by the treating practitioner based on contemporaneous clinical notes documenting patient-reported experiences at the final treatment session and at the follow-up visit. Case 1 reported substantial reduction in pain during daily activities including cutting and lifting and expressed satisfaction with the non-surgical outcome given her previous failed conservative treatment. Case 2, who had been strongly advised to undergo arthroscopic debridement, reported meaningful improvement in grip strength and reduction in pain during tool operation and expressed relief at avoiding surgical intervention. Case 3 reported resolution of pain sufficient to permit return to work as a chef prior to completion of the treatment course and sustained improvement at long-term follow-up.

## 3. Discussion

This case series describes three patients with refractory LE who achieved clinically meaningful improvements in pain and function following a structured multicomponent ultrasound-guided pharmacopuncture protocol. Although MTJ-targeted hydrodissection pharmacopuncture was the central and most frequently administered component, the relative contribution of each treatment stage to the observed outcomes cannot be determined from this case series design.

The MTJ is an anatomically distinct zone at which the mechanical loads generated by muscle contraction are transmitted to the tendon, rendering it susceptible to repetitive microtrauma and degenerative change [8]. Consistent with this, all patients in the present series demonstrated focal tenderness on probe compression at the MTJ in addition to the CET enthesis. While this finding is consistent with MTJ involvement as a contributing pain source, it does not constitute independent confirmation of the MTJ as a distinct pain generator, given the absence of a diagnostic block or validated provocation procedure. MTJ tenderness is therefore reported as an exploratory clinical observation that informed the selection of the MTJ as the primary treatment target. The present cases extend this concept by demonstrating that ultrasound-guided pharmacopuncture at the MTJ, combined with sequential hydrodissection, can yield clinically meaningful outcomes in patients who had not responded to conventional enthesis-centered treatments. Rosales et al. proposed the MTJ as an advantageous infiltration site for ultrasound-guided injection, citing benefits including cost-effectiveness, precise needle placement, and absence of radiation exposure [8]. The authors note several potential considerations, including the risk of local hematoma, high operator dependency, and the requirement for dedicated imaging equipment; particular care should be taken to identify relevant anatomic landmarks, maintain aseptic technique, and avoid injection in close proximity to tendon tears.

The pharmacopuncture agent used in the present series merits consideration. Numerous in vitro studies have demonstrated potential cytotoxic effects of local anesthetics at clinically relevant concentrations on human mesenchymal stem cells [17]. Although local anesthetics were not used in the present protocol, this cytotoxicity evidence informed the selection of placenta pharmacopuncture as the injectate for Stage 1, given its tissue-regenerative profile and the absence of documented cytotoxic effects on tenocytes. Previous Korean clinical evidence indicates that adverse events associated with pharmacopuncture are infrequent, generally mild, and transient [18]. In the present series, no systemic adverse events or clinically significant local complications were observed following the MTJ-targeted pharmacopuncture procedure.

Jungsong-ouhyul (Ouhyul/Eohyeol) pharmacopuncture has been used in Korean clinical studies predominantly for musculoskeletal pain conditions, particularly blood stasis-associated pain, low back pain, and acute traumatic pain, with improvements in pain-related outcomes documented across several clinical reports [19]. The same review noted no serious treatment-related adverse events, although the authors acknowledged that adverse-event reporting was inconsistent across the included studies [19]. Placenta pharmacopuncture, prepared from hydrolyzed human placenta, is reported to contain diverse bioactive constituents—including growth factors, cytokine-related substances, nucleic acid derivatives, vitamins, and hormone-related compounds—and has been used with the therapeutic aim of supporting tissue repair and regeneration [20].

The two-stage hydrodissection technique employed herein—targeting both the inferior and superior aspects of the MTJ—was designed to achieve mechanical dissection of peri-junctional adhesions while distributing the pharmacopuncture agent across the full extent of the MTJ. This sequential approach draws conceptual parallels with the staged hydrodissection framework described for chronic Achilles tendinopathy [21], in that both protocols target distinct tissue planes in a stepwise manner; however, the anatomical structures, needle trajectories, and pathological targets differ substantially between the two conditions, and the adaptation to the lateral elbow context is considered exploratory.

Each stage of the protocol was designed to target distinct hypothesized pathophysiological mechanisms of chronic LE, as detailed below, although these mechanisms were not directly assessed in the present series and remain speculative.

Stage 1, performed routinely at every visit for a total of 10 sessions, targeted the MTJ, which is densely populated with nociceptors, and was hypothesized to modulate nociceptive signaling and address myofascial trigger points.

Stage 2, introduced during the first two visits (2 sessions total), was hypothesized to reduce peri-neural tension involving the posterior antebrachial cutaneous nerve and attenuate peripheral sensitization through hydrodissection of the deep fascial plane.

Finally, Stage 3, administered during the ninth and tenth visits (2 sessions total), utilized dry needling at the CET enthesis, hypothesized to promote fibroblastic proliferation and structural remodeling at the bone–tendon junction. This multi-targeted, structured approach hypothesizes comprehensive management addressing both neurogenic pain and structural tendinopathy.

All three cases demonstrated within-patient PRTEE reductions exceeding the MCID threshold of 37% [16]. While this indicates a magnitude of change considered clinically meaningful at the individual level, it does not constitute evidence of treatment efficacy in the absence of a comparator group. Furthermore, sonographic improvements were maintained at follow-up assessments conducted 103 to 270 days after treatment completion (Table 1 and Table 2).

The relatively smaller improvement observed in Case 2 (−47.9%) compared with Cases 1 and 3 (−79.7% and −79.1%, respectively) warrants discussion. Case 2 presented with the highest baseline PRTEE score (73 points) and the most severe clinical course, having been referred for arthroscopic surgery prior to presentation. The more limited response may reflect a greater degree of structural tendon disruption or a more advanced degenerative state. Nonetheless, even in this challenging case, the magnitude of improvement exceeded the MCID, and the patient avoided surgical intervention.

Several alternative explanations for the observed improvements warrant consideration. In the absence of a control group, spontaneous natural recovery and regression to the mean cannot be excluded, as patients typically present at peak symptom severity. Placebo effects, patient expectation, and the concurrent eccentric exercise program may also have contributed to the observed outcomes. These factors substantially limit causal interpretation of the present findings.

Several limitations of the present report should be acknowledged. As a retrospective observational case series with a small number of cases, causal inference cannot be drawn, outcome assessors were not blinded, and generalization is not possible. The contribution of individual treatment components—including rehabilitation exercises and potential changes in occupational loading—cannot be disentangled. Clinical outcomes were assessed only at baseline and treatment completion; maintenance of improvement beyond this point cannot be confirmed in the absence of follow-up PRTEE data. Sonographic outcomes were assessed qualitatively, follow-up duration was unequal across cases, and adverse-event monitoring relied on verbal inquiry without a standardized instrument.

The eccentric exercise program represents a significant time-varying co-intervention. Sonographic changes observed at follow-up may reflect the cumulative effects of the pharmacopuncture protocol, the exercise program, natural tendon remodeling over time, or a combination thereof. The relative contribution of exercise to the observed outcomes cannot be determined from this case series.

Future prospective studies incorporating standardized treatment protocols, control arms, and quantitative sonographic outcome measures are warranted to validate the efficacy and elucidate the mechanism of MTJ-targeted pharmacopuncture in LE.

## 4. Conclusions

This case series provides preliminary evidence that a structured multicomponent ultrasound-guided pharmacopuncture protocol may represent a feasible non-surgical approach for patients with refractory LE. The consistent improvements in PRTEE scores and sonographic findings across three cases with varying degrees of tendon pathology generate hypotheses regarding the potential of this approach. However, the observed improvements cannot be attributed to any single component. Controlled prospective studies are warranted to establish the efficacy, standardize the pharmacopuncture protocol, and elucidate the underlying mechanisms of MTJ-targeted intervention in lateral epicondylalgia.

## Figures and Tables

**Table 1:** Baseline Patient Characteristics and Clinical Timeline.

	Duration of Symptoms	Diagnosis	Prior Conservative Treatments	Concurrent Medications	Date of Initial Visit	Date of Treatment Completion	Follow-Up Sonography
Case 1 (62/F)	1.5 yrs	Partial tear of CET	Triamcinolone injection	None	14 April 2025	7 May 2025	6 August 2025 (114 days)
Case 2 (54/M)	2 yrs	Partial tear of CET	Corticosteroid injection, NSAIDs, physical therapy	None	18 July 2022	10 August 2022	21 November 2022 (103 days)
Case 3 (48/M)	3 yrs	CET tendinopathy	Prolotherapy injection	Antihypertensive agent	11 June 2021	30 June 2021	8 March 2022 (270 days)

Footnotes—Baseline: date of initial visit and first PRTEE administration prior to treatment. Treatment completion: date of tenth session. Follow-up sonography: date of post-treatment ultrasound assessment.

**Table 2:** PRTEE scores at baseline and post-treatment completion.

	Baseline PRTEE Score	Post-Treatment PRTEE Score	Score Change	% Improvement
Case 1 (62/F)	64	13	−51	−79.7
Case 2 (54/M)	73	38	−35	−47.9
Case 3 (48/M)	55	11.5	−43.5	−79.1

Baseline PRTEE: administered at initial visit prior to first treatment. Post-treatment PRTEE: administered at the tenth (final) session. No follow-up PRTEE data are available for this series.

**Table 3:** Individual treatment timeline for all patients.

	Case 1	Case 2	Case 3
Initial visit Session 1	14 April 2025	18 July 2022	11 June 2021
Exercise initiation (Session 9)	April 2025	August 2022	June 2021
Treatment completion (Session 10)	7 May 2025	10 August 2022	30 June 2021
PRTEE (baseline)	14 April 2025	18 July 2022	11 June 2021
PRTEE (post-treatment)	7 May 2025	10 Aug 2022	30 June 2021
Follow-up sonography	6 August 2025	21 November 2022	8 March 2022

## Data Availability

The original contributions presented in the study are included in the article. Further inquiries can be directed to the corresponding author.

## Supplementary Materials

The following supporting information can be downloaded at https://www.mdpi.com/article/10.3390/diagnostics16172755/s1. Supplementary File S1—CARE-checklist. Supplementary File S2—Real-time ultrasound-guided procedure video.

## Abbreviations

The following abbreviations are used in this manuscript:

LE	Lateral epicondylalgia
CET	Common extensor tendon
MTJ	Myotendinous junction
PRTEE	Patient-Rated Tennis Elbow Evaluation
ECRB	Extensor carpi radialis brevis
EDC	Extensor digitorum communis
SUP	Supinator
R	Radius
US	Ultrasonography
APCA	Alliance for Physician Certification & Advancement
RMSK	Registered in Musculoskeletal Sonography
MCID	Minimal Clinically Important Difference
