# Supplementary material for: Ultrasound-Guided Pharmacopuncture Targeting the Myotendinous Junction for Refractory Common Extensor Tendinopathy: A Case Series
Source: Diagnostics (Basel). 2026 Aug 28;16(17):2755. doi: 10.3390/diagnostics16172755 (PMC13564665; doi:10.3390/diagnostics16172755)
Supplement: Supplementary file 1 [file diagnostics-16-02755-s001.zip › Supplementary file S1 - CARE-checklist.pdf]

CARE case report guidelines

CARE Checklist of information to include when writing a case report

(cc) BY-NC-ND

Topic	Item	Checklist item description	Reported on Line
Title	1	The diagnosis or intervention of primary focus followed by the words “case report”	page 1 Line 0
Key Words	2	2 to 5 key words that identify diagnoses or interventions in this case report, including "case report"	page 1 Line 22
Abstract (no references)	3a	Introduction: What is unique about this case and what does it add to the scientific literature?	page 1 Line 28
	3b	Main symptoms and/or important clinical findings	page 1 Line 33
	3c	The main diagnoses, therapeutic interventions, and outcomes	page 1 Line 28
	3d	Conclusion—What is the main “take-away” lesson(s) from this case?	page 1 Line 37
Introduction	4	One or two paragraphs summarizing why this case is unique (**may include references**)	page 2 Line 84
Patient Information	5a	De-identified patient specific information.	page 2 Line97-114
	5b	Primary concerns and symptoms of the patient.	page 2 Line97-114
	5c	Medical, family, and psycho-social history including relevant genetic information	page 2 Line97-114
	5d	Relevant past interventions with outcomes	page 2 Line97-114
Clinical Findings	6	Describe significant physical examination (PE) and important clinical findings.	page 2 Line116-117
Timeline	7	Historical and current information from this episode of care organized as a timeline	page 2 Line119-120
Diagnostic Assessment	8a	Diagnostic testing (such as PE, laboratory testing, imaging, surveys).	page 2 Line116-117
	8b	Diagnostic challenges (such as access to testing, financial, or cultural)	page 6 Line239-243
	8c	Diagnosis (including other diagnoses considered)	page 4 Line152
	8d	Prognosis (such as staging in oncology) where applicable	not applicable
Therapeutic Intervention	9a	Types of therapeutic intervention (such as pharmacologic, surgical, preventive, self-care)	page4 Line 154-174
	9b	Administration of therapeutic intervention (such as dosage, strength, duration)	page4 Line 154-160
	9c	Changes in therapeutic intervention (with rationale)	page4 Line 162-174
Follow-up and Outcomes	10a	Clinician and patient-assessed outcomes (if available)	page5 Line 175-222
	10b	Important follow-up diagnostic and other test results	page5 Line 175-222
	10c	Intervention adherence and tolerability (How was this assessed?)	page5 Line 191, 210
	10d	Adverse and unanticipated events	page5 Line 193, 212
Discussion	11a	A scientific discussion of the strengths AND limitations associated with this case report	page6 Line 224-286
	11b	Discussion of the relevant medical literature **with references**	page6 Line 224-286
	11c	The scientific rationale for any conclusions (including assessment of possible causes)	page7 Line 267-274
	11d	The primary “take-away” lessons of this case report (without references) in a one paragraph conclusion	page7 Line 289-293
Patient Perspective	12	The patient should share their perspective in one to two paragraphs on the treatment(s) they received	page6 Line 216-222
Informed Consent	13	Did the patient give informed consent? Please provide if requested	Yes ☑ No ☐
